# The connection to the public’s preferred sports analysis and physical education curriculum

**DOI:** 10.1371/journal.pone.0264032

**Published:** 2022-03-16

**Authors:** Yong-Wook Kim, Jinyoung Han, Kyungtae Jang, Minsam Ko, Jaewoo Park, Seungyup Lim, Jin-Young Lee

**Affiliations:** 1 Department of Human-Computer Interaction, Hanyang University, Ansan, South Korea; 2 Department of Applied Artificial Intelligence, Sungkyunkwan University, Seoul, South Korea; 3 Department of Culture and Tourism, Komazawa Women’s University, Tokyo, Japan; 4 Division of Sport Science, Hanyang University, Ansan, South Korea; 5 Division of Global Sport Studies, Korea University, Sejong, South Korea; Universiti Malaysia Terengganu, MALAYSIA

## Abstract

People have their favorite type of sport, but such preferences tend to be shared for nearly a lifetime. How this preference persists remains inconclusive; hence, this study attempts to determine why people have different viewpoints on sports. It is reasonable to infer that these differences arise from differences in culture, occupation, and race. Therefore, we collected the following data and conducted research in Korea, the United States, and Japan, countries with various differences. The types of sports that people play were collected through surveys and comparisons among sports networks. Namely, “Sport Classification,” “The K-12 Physical Education System (textbooks),” “Survey (actual physical activity),” “Simple Notification Service (SNS) Activity” have been examined to deduce the reason why any particular sport is played. Firstly, Korea, the United States, and Japan conduct different physical education courses. Hence, the results affect people’s preferences. Secondly, what people post on SNS and their actual physical activities are different. Thirdly, the degree of connection between sports-type varied as well. Lastly, sports that serve the purpose of being regarded as hubs among sports-type were common in Korea, the United States, and Japan.

## Introduction

Michael Jordan, the greatest player in NBA, announced his retirement from professional basketball in October 1993. The retirement of the player considered as the “Greatest of All Time” in NBA history was unexpected. The next year, however, the world could witness him involved in another sport. He signed a contract with Chicago White Sox and spent the season as a baseball player in Birmingham Barons, the double-A team in MiLB. It was an unimaginable event where an athlete played both professional basketball and baseball, two seemingly unrelated sports. Ultimately, Jordan finished the 1994 season with a 0.202 batting average, 3 home runs, 51 runs batted in, 0.289 On Base Percentage, and .556 On Base Percentage Plus Slugging Percentage in the 127 games with double-A [1].

However, the event demonstrated that a player with excellent physical ability could gain a prime opportunity by transitioning to another sport. Unlike Jordan, there is an athlete who succeeded in sports career transition. He is SeungHoon Lee, who acquired a gold medal in speed skating at the 2018 Winter Olympics–Men’s Mass Start. He also won a gold medal in the 2008 World Short Track Speed Skating Championships– 3000m [2]. Although speed skating and short track share a common feature in that the players skate on ice, they are different sports in terms of their dissimilar stadium sizes and skating postures. In comparison with track-and-field, it can be similar to the situation where a sprinter challenges a marathon runner. Nonetheless, it is amazing to witness the world’s best performances equally in such distinctly dissimilar sports.

Some cases of famous sports stars are as follows. Watching Rafael Nadal, the tennis player, and Cristiano Ronaldo, the football player, playing tennis with football, it was evident in the performance that Rafael Nadal has a remarkable ability in football. It is said that he was a football player in his childhood and a fan of Real Madrid, the professional football team in La Liga [3,4]. Moreover, Tiger Woods, the golf player, is known as the best friend of Serena Williams, the tennis player. For that reason, it is not awkward to witness him on the spectator’s seat of a tennis court at the US Open, and his tennis ability is believed to be above amateur’s level [5]. It is widely known that many players enjoy not only their main sports but also other types of sports. However, it is often mistaken to think that players are not able to learn the sports apart from their main ones and even afford to enjoy them. The mistake is often derived from a thought that if a player wishes to be the greatest in the world, it is necessary to conduct the training related only to his or her main sport. The fact is that the world’s best players are enjoying multiple sports as hobbies. In other words, it can be understood that athletes enjoy multiple sports rather than only one sport.

If so, which patterns govern how people enjoy sports? Which sports do people, who normally enjoy baseball, play additionally? Do people enjoy golf like tennis? If people enjoying golf choose to play tennis, what connection leads people to make such a choice? Does the choice result from the experience of the K-12 Physical Education System? To find answers to these questions, such notions should be evaluated based on people’s experiences. Sport types are important because they affect lifelong exercise activities in the school curriculum where exercise is first learned. Therefore, we analyzed the K-12 Physical Education System to see how people’s learning experiences affect their lifelong exercise activities.

## Literature review

Spectator sports, which are popular among enthusiasts of professional sports and participation sports, differ in terms of leisure and life satisfaction [6–9]. Concerning spectator sports, football is popular in the USA, Korea, and Japan, countries known to prefer baseball [10]. Moreover, the result of the survey on people’s physical activities in Korea, Japan, and the USA is illustrated in Table 1, representing the varying preferences of sports in each nation. Upon close examination, it is revealed that people in Korea like walking (40.8%), mountain climbing (23.2%), and fitness (11.9%). In contrast, data from Japan denotes a preference towards walking (57.0%), fitness (12.9%), and gymnastics (12.4%). Further, fitness sports (66.0%), outdoor sports (59.2%), and individual sports (45.3%) were ranked in ascending order in the USA (Table 1). The difference in the preferences of sports across countries is caused by various factors, such as culture and geography [10]. One of the main factors accounting for the differences can be the K-12 Physical Education System. This correlation is assumed because the K-12 Physical Education Systems in Korea, the USA, and Japan are different as adults tend to play accustomed sports through the aforementioned system [11].

**Table 1:** Popularity of community sports in Korea, Japan, and the USA.

Sports	Korea [12]	Japan [13]	Sports	USA [14]
Aerobics	2.7%	0.1%	fitness sports	66.0%
Badminton	7.8%	3.1%		
Baseball	2.3%	5.9%		
Basketball	5.3%	1.7%	individual sports	45.3%
Billiards	8.3%	0.1%		
Bowling	7.1%	4.7%		
Cycling	8.9%	10.9%	outdoor sports	59.2%
Dancing	1.4%	2.5%		
Fencing	0.7%	0.1%		
Fishing	4.1%	4.5%	racquet sports	13.0%
Fitness	11.9%	12.9%		
Football	8.9%	4.05%		
Golf	4.0%	11.0%	team sports	22.6%
Gymnastics	9.4%	12.4%		
Martial arts	1.7%	0.1%		
Mountain climbing	23.2%	3.9%	water sports	13.7%
Ping pong	3.5%	3.2%		
Rope skipping	7.7%	2.2%		
Swimming	8.3%	5.2%		
Tennis	1.4%	3.8%		
Track and field	3.3%	12.2%		
Volleyball	1.5%	1.9%	winter sports	7.1%
Walking	40.8%	57.0%		
Yoga	6.3%	6.3%		

Ministry of Culture, Sports and Tourism-Korea. 2018 [12]; Physical Activity Council, 2019 [14]; Japan Sports Agency, 2019 [13].

There are two main ways to select sports that are covered in the school curriculum. First, there is a standard for selecting basic sports for the physical development of students. Students have different athletic abilities depending on their physical age. Basic exercises that meet each age standard should be performed. These exercises help students develop their bodies. Second, there is a standard for selecting various sports to attract students’ interest. Basic events such as running and walking are difficult to attract students’ interest. It is difficult to create a sense of cooperation that is the basis of social life. Therefore, to integrate cooperation into physical education, various sports, such as group sports, should be utilized to attract students’ interest. The physical education curriculum should consist of ’Basic exercises’ and ’Fun sports’ in harmony. These two conditions are contrary to each other. How the two are harmoniously distributed leads to either the success the or failure of physical education.

The various sports constituting the K-12 Physical Education System are integral because they influence individuals’ lifelong sports activities. The emphasis on the physical education system in each country is stated below. The K-12 Physical Education System in Korea is based on body movement, which consists of health management ability, physical training ability, competition performance ability, and body expression ability. Through this process, participants are expected to learn various sports [15]. Japan’s K-12 Physical Education System aims to improve health and physical strength by understanding basic movements of exercise and solving fundamental tasks [16–18]. The K-12 Physical Education System in the USA aims to improve various skills, knowledge, social behavior, and recognition of the value of actual physical activity for health, enjoyment, challenge, self-expression, and/or social interaction [19].

As mentioned above, studies on the influence of the K-12 Physical Education System on lifelong sports activities have been reported in various ways [20,21]. A study demonstrating that differences in culture, geography, and economy has impact on sports activities was also reported [11,22]. Various studies have been conducted on the recognition of products and consumers in the marketing research field of public goods. In determining a location for a store and displaying products, the relationship between selling products and customers’ preferences has been investigated in locations such as department stores [23,24].

Thus, to explain the sports network, which is the objective of this study, connections between sports should be analyzed. Studies on the analysis of correlations among sports are rare except in research that classifies and categorizes the characteristics of sports and then determines their positions [25,26]. For example, a variety of factors can be correlated, such as popularity, stadium, strategies, and players’ movement skills. For an accurate analysis of correlations among sports, it is necessary to draw various sports networks, decipher their meanings, and explain them with great insight. It is also imperative to adapt to a means of network analysis that thoroughly examines the K-12 Physical Education System as well as the actual physical activity and Simple Notification Service (SNS) activities required to conduct such a study.

Generally, social network analysis can be explained by identifying the mutual connections between the subjects in the study group [27]. The connection mentioned here can be classified largely into three categories–“human vs. human,” “object vs. object,” and “event vs. event” [28]. The way to intuitively identify these connections is to visualize and observe the patterns of the connections. The visualized outcomes are composed of nodes and edges. A node refers to the subjects of these connections, such as humans, events, and objects. Edge happens to be a connection of the Node, which can have either directivity or only a simple connection without directivity according to the properties of the connections. We can intuitively perceive whether our relationship with others is good, bad, hostile, or intimate. As the perception is enabled by our experience, it has been regarded as unquantifiable. Nevertheless, if it is connected with the directivity-given edge, the connected networks can visualize mutual relations such as those among groups, organizations, and events. Therefore, the characteristics of the networks can be established. Besides, the deduction of properties and meanings of the networks can also be enabled by identifying the measured outcome in quantified figures [29,30].

## Materials and methods

This study method includes a survey of Koreans, Japanese, and Americans. The contents of the survey include the subject’s physical activity and preference for sports events. The number of survey subjects was set at 350 each according to the survey method of previous study [31]. In addition, detailed explanations of the survey contents were notified to the survey subjects and consent was obtained for the survey. This study was conducted with the approval of IRB.

This study aims to analyze the correlations of sport types. The sports types that people play have been collected by surveys and the comparisons between sports networks–“Sport Classification,” “The K-12 Physical Education System,” “Survey (actual physical activity),” and “SNS Activity” to deduce the reason why they play the sport. Based on the connections between sports types constructed in each field, the associative sports networks were drawn. This study also verifies the characteristics of sports networks by investigating their similarities and examining whether they vary by country (i.e., Korea, the USA, and Japan). Notably, Korea and Japan were selected due to their close regional and cultural similarities, and the United States was selected due to its great influence on the political culture of Korea and Japan after World War II.

To achieve the goal of this study, data were collected through the process described below. A total of 24 sports types were selected as subjects for the analysis before data collection: aerobics, badminton, baseball, basketball, billiards, bowling, cycling, dancing, fencing, fishing, fitness, football, golf, gymnastics, martial arts, mountain climbing, ping pong, skipping rope, swimming, tennis, track and field, volleyball, walking, and yoga. The selection of the sports types was based on “A Survey on the Participation in Sports Activities in Korea,” which was conducted through an expert meeting (i.e., five scholars from the discipline of sport studies).

First, the “Sport Classification” was carried out through expert surveys. The experts who participated in the survey were a group of six people with Ph.D degrees in sports. The expert group held three meetings over the course of two months to select survey items. The 41 categories (Table 2) classified by sports characteristics were selected during the experts’ meeting, and the data were collected after receiving the response from the experts to identify the connections among sports types. The collected data was arranged multidimensionally according to 41 characteristics distributed among 276 pairs of Sport types, and the distance between the types was measured to identify the connections between the 24 types. Responses were provided on a 5-point Likert scale. In addition, the distance between the types was measured using Jaccard’s coefficient. Cronbach’s alpha value was checked to verify the reliability of all 41 questions, and values between 0.822 and 0.929 were found for all questions.

**Table 2:** Sports characteristics.

Sports Characteristics
1. Is the main exercise place indoors? 2. Do you use limited venues (fields)? 3. Are the sports venues being moved? 4. Do we follow a set course when moving? 5. Do you usually use your hands? 6. Do you usually use your feet? 7. Do you use your whole body? 8. Do you use running motions? 9. Do you use walking motions? 10. Do you use rolling motions? 11. Do you use body rotation? 12. Do you use the arm rotation movement? 13. Do you use the throwing motion? 14. Do you use the hanging motion? 15. Do you use jump actions? 16. Do you use dribble movements? 17. Do you use head movements? 18. Do you use the ball rolling motion? 19. Do you use the kick motion to kick the ball? 20. Do you use your hand to receive the ball? 21. Do you use hand-punching movements? 22. Do you use equipment to hit the ball? 23. Do you use the wielding motion of the equipment in your hand? 24. Does it involve frequent physical contact with the other party? 25. Do you use actions that pose threats to the other party? 26. Is it an exercise that you usually do alone? 27. Is it mainly a team sport? 28. Is it an exercise that has an opponent? 29. Is it an exercise that usually records scores? 30. Is it primarily a time-measuring exercise? 31. Is it an exercise that uses equipment or instruments a lot? 32. Is it an exercise that usually uses a ball? 33. Is it an exercise that requires wearing protective gear to protect the body? 34. Is it an exercise that requires sports equipment? 35. Is it an exercise that uses goalposts? 36. Is it an exercise that uses a net? 37. Is it an exercise that mainly uses hand-held equipment? 38. Is it an aerobic exercise? 39. Is it a muscle workout? 40. Do you need music when you exercise? 41. Is equipment essential to partake in the exercise?

For the analysis of the K-12 Physical Education System, the documents of the curricula in Korea, the USA, and Japan were collected as exhibited in Table 3. The documents of the curriculum in Korea include 10,303 words and 124 pages, and the ones in the USA comprise 50,174 words and 136 pages. Japan’s curriculum includes 26,637 words and 913 pages. The paragraphs in each document were extracted to identify the connections between sports types that appeared in each curriculum. The numbers of paragraphs are classified as 934 paragraphs in Korea, 4,674 paragraphs in the USA, and 2,134 paragraphs in Japan. The sports types are connected through their classification into pairs of sport types that appear in the same paragraph.

**Table 3:** Textbooks.

Country	K-12 Physical Education Systems			
	Textbooks	Page	Paragraphs	Words
Korea	2015 Physical Education Course [15]	124	934	10,303
USA	National Standards & Grade-Level Outcomes for K–12 Physical Education [19]	913	2,134	26,637
Japan	Elementary School Physical Education Course [16] Middle School Physical Education Course [17] High School Courses Physical Education Course [18]	136	4,674	50,174

Ministry of Education-Korea, 2015 [15]; Couturier L, Stevie C, Shirley HH, 2014 [19]; Ministry of Education, Culture, Sports, Science and Technology-Japan, 2017a [16]; Ministry of Education, Culture, Sports, Science and Technology-Japan, 2017b [17]; Ministry of Education, Culture, Sports, Science and Technology-Japan, 2017c [18].

Third, surveys (actual physical activity) were conducted in Korea, the USA, and Japan to identify the types of sports that people actually play. Third, surveys (actual physical activity) were conducted in Korea, the USA, and Japan to identify the types of sports that people actually play. A total of 1,662 people participated in the survey, with 547 people from the United States, 753 people from Korea, and 362 people from Japan participating. The surveys were conducted simultaneously in the three countries over the course of one month in May 2019. Convenience sampling was utilized as the survey method. Face-to-face surveys were adapted in Korea and Japan, while an online survey was used in the USA. The general features of the collected data are shown in Table 4. The connections between sports types were drawn by receiving responses to the survey questions inquiring about the sports played together. If two physical activities were responded to, they were connected once, and if three answers were retrieved, they were connected after being classified into each pair of sport types. The survey results verified that the number of people participating in more than two physical activities was 264 (60.55%) in Korea, 505 (78.54%) in the USA, and 172 (55.66%) in Japan. The relationship between occupation, age, income level, and sports events collected in the survey will be further analyzed in a follow-up study that has been developed.

**Table 4:** Sample characteristic (actual physical activity).

Sample Characteristic		Korea	USA	Japan
Gender	Male	248	307	178
	Female	299	446	184
Job Classification	Student	263	28	152
	Businessman	129	37	102
	Public servant	2	63	1
	Management	10	103	11
	Professional	40	247	34
	Self-employed	32	117	27
	Homemaker	42	69	17
	Others	29	89	18
Age Group	21~30 years	315	161	148
	31~40 years	110	242	102
	41~50 years	74	165	68
	51~60 years	40	108	30
	Over 60 years	8	77	14
Monthly Household Income	under 2,000 dollars	176	164	124
	2,001~4,000 dollars	163	283	102
	4,001~6,000 dollars	92	174	48
	6,001 dollars or more	116	132	88
Exercise Frequency (per Week)	1 time	94	31	71
	2 times	56	93	58
	3 times	56	176	39
	4 or more times	74	288	49
	I don’t exercise.	111	110	53
	I exercise irregularly.	156	55	92
Exercise Frequency (per session)	Less than 30 minutes	120	152	77
	30~60 minutes	146	370	88
	60~120 minutes	65	94	51
	Over than 120 minutes	24	8	24
	I don’t exercise.	111	110	53
	Different from time to time	81	19	69
The types of Physical Activity You Do	I don’t exercise.	111	110	53
	1 type	172	138	137
	2 types	110	169	88
	3 types	94	150	61
	4 or more types	60	186	23
Total		547	753	362

Finally, the posting activities of the SNS were used for data analysis, wherein the collected posts related to physical activities were uploaded on Instagram. The collection was facilitated by web crawling using keywords related to 24 sports types on Instagram. Web crawling is a method of automatically collecting information posted online by creating an Internet bot program. Through this process, sports types were collected as data from posts uploaded by SNS users. The collected data are presented in Table 5. The connections between the sports types in the SNS activities were enabled by searching for posts written by the same SNS user. If two physical activities were responded to, they were connected once, and if more than three activities were undertaken, they were connected after being classified into each pair of sports types.

**Table 5:** SNS (Instagram).

Sports	Korean	English	Japanese
Aerobics	70,510	99,067	10,249
Badminton	120,352	120,477	78,324
Baseball	92,352	82,601	71,188
Basketball	102,555	80,570	85,220
Billiards	96,122	93,982	75,018
Bowling	99,954	75,142	58,415
Cycling	130,627	89,122	71,998
Dancing	40,892	78,057	102,359
Fencing	191,595	74,955	130,521
Fishing	117,441	68,557	74,811
Fitness	146,926	84,322	112,573
Football	82,275	89,616	79,598
Golf	140,838	79,074	84,726
Gymnastics	167,595	76,228	104,068
Martial arts	101,592	77,024	161,169
Mountain climbing	110,028	92,609	84,705
Ping pong	97,942	85,236	79,078
Rope skipping	121,717	92,353	15,997
Swimming	115,560	67,610	91,642
Tennis	118,433	79,541	84,745
Track and field	111,066	171,814	97,354
Volleyball	98,997	91,415	82,336
Walking	112,044	67,415	65,907
Yoga	156,496	105,706	144,332
Total	2,743,909	2,122,493	2,046,326
	6,912,728		

The collected data displayed above draws sports networks to identify the relations between sports. We introduce the notion of the “Sports Network” as an undirected graph G = (N, E), where a node indicates a sport and an edge indicates the relation between two sports. To see the similarity between “Sports Networks,” both “Edge Overlaps” and “Node Overlaps” were measured. “Edge Overlaps” was measured using the method of “Jaccard’s coefficient” and the method of “Pearson’s correlation coefficient” was used to measure “Node Overlaps.” While examining “Edge Overlaps” and “Node Overlaps” during the network analysis, the similarity between the two networks can be identified in figures. The differences recognized intuitively in the network graphs can be seen quantitatively through “Edge Overlaps” and “Node Overlaps” [32–34]. This study uses “R version 3.6.0,” a statistical program, to analyze the data and “Gephi 0.9.2” to visualize the graphs.

## Results and discussion

### Textbook-oriented differences according to the countries

**Fig 1:**
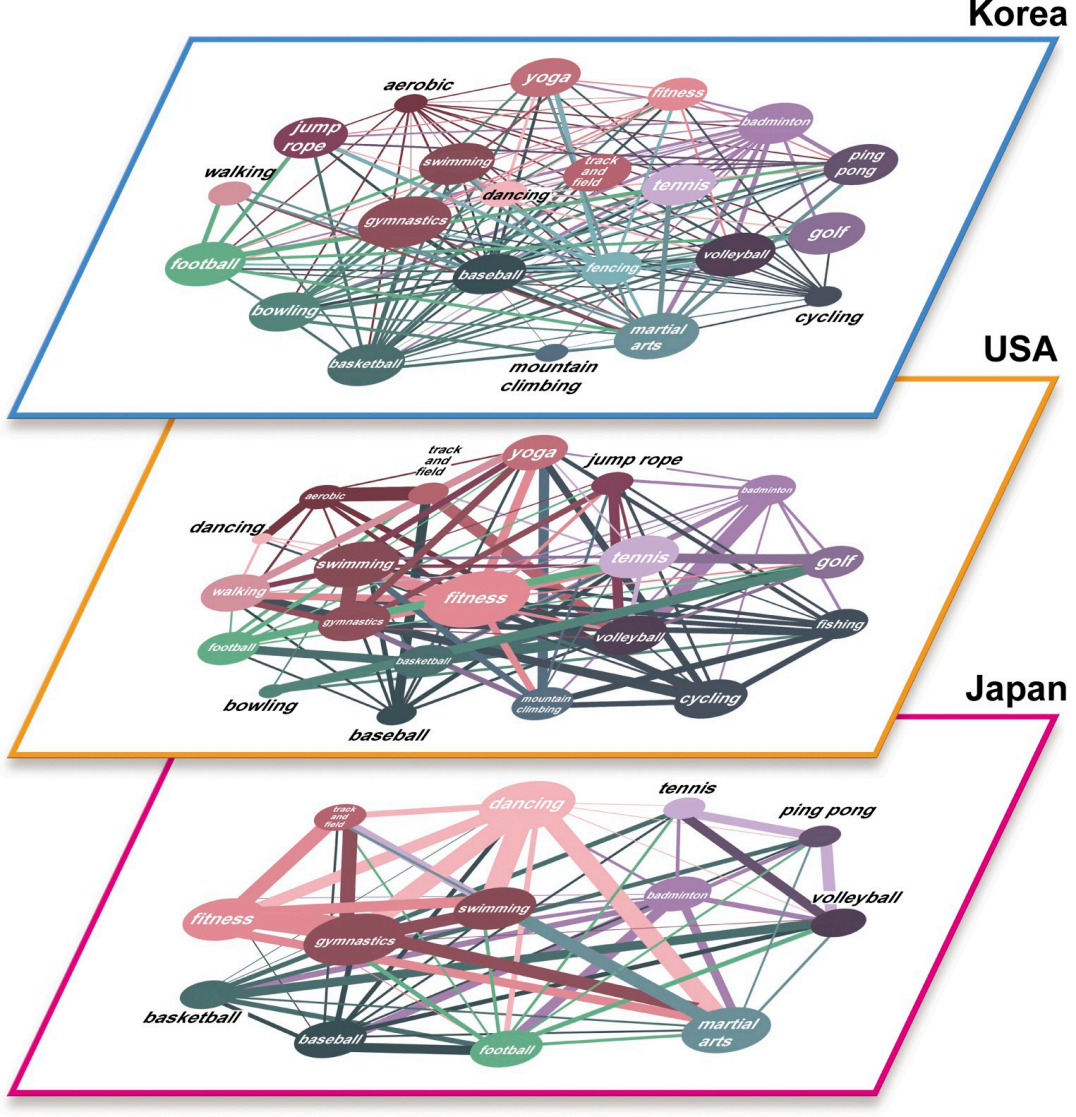
Textbooks.

The comparative analysis that draws sports networks of “The K-12 Physical Education System,” “Survey (actual physical activity),” and “SNS Activity” was carried out to see the differences in Korea, the USA, and Japan. The sports networks of the K-12 Physical Education System in each country are shown in Fig 1. As depicted in the graph, it is noticeable that the countries have distinct networks. In Table 6 where the detailed comparisons between the countries are highlighted, the rate of the similarity of “the K-12 Physical Education System” in Korea and Japan is evaluated as low, with ‘Node Overlaps’ of 0.349***. Moreover, the USA and Japan have a low rate of similarity indicated by the ‘Node Overlaps’ of 0.134*. Lastly, Korea and the USA are verified to have no similarity. Contrariwise, the similarity between Korea and Japan was thought to be due to geographical and cultural similarities, as well as their curricula, which are dominantly run by the government. The results derived from the USA, on the other hand, are considered to be caused by the fact that only brief guidelines for the curriculum are suggested in there [35,36]. Consequently, the curricula in Korea and Japan have no impact on actual physical activities, while the USA does denote that capability to influence certain elements. This effect persists because of the differences in each country’s curricula.

**Table 6:** Network overlaps (textbook vs. country-by-country).

*X*	*Y*	Edge Overlaps (Jaccard’s coefficient)	Node Overlaps (Pearson’s Correlation coefficient)
Korea textbook	USA textbook	0.10840738	0.09728171
Korea textbook	Japan textbook	0.080288147	0.349266***
USA textbook	Japan textbook	0.034182492	0.1346034*

* *p* < .05

** *p* < .01

*** *p* < .001.

### Connections between textbooks and actual physical activity

The differences are evident when comparing the results of ‘The K-12 Physical Education System” and “Survey (actual physical activity)” in each country. Specifically, for the USA, a low similarity is indicated, which is 0.298***, as revealed in Table 7. Nonetheless, no similarity appears between Korea and Japan. Consequently, the sports networks of “The K-12 Physical Education System” and “Survey (actual physical activity)” are shown to be different in all three countries, Korea, the USA, and Japan. This dissimilarity means that the sports activities dealt with in “The K-12 Physical Education System” did not mimic actual sports activities.

**Table 7:** Network overlaps.

*X*	*Y*	Edge Overlaps (Jaccard’s coefficient)	Node Overlaps (Pearson’s Correlation Coefficient)
Korea textbook	Korea survey	0.119293298	-0.009021564
USA textbook	USA survey	0.11139786	0.2983137***
Japan textbook	Japan survey	0.054412987	0.0001298196

* *p* < .05, ** *p* < .01

*** *p* < .001.

**Fig 2:**
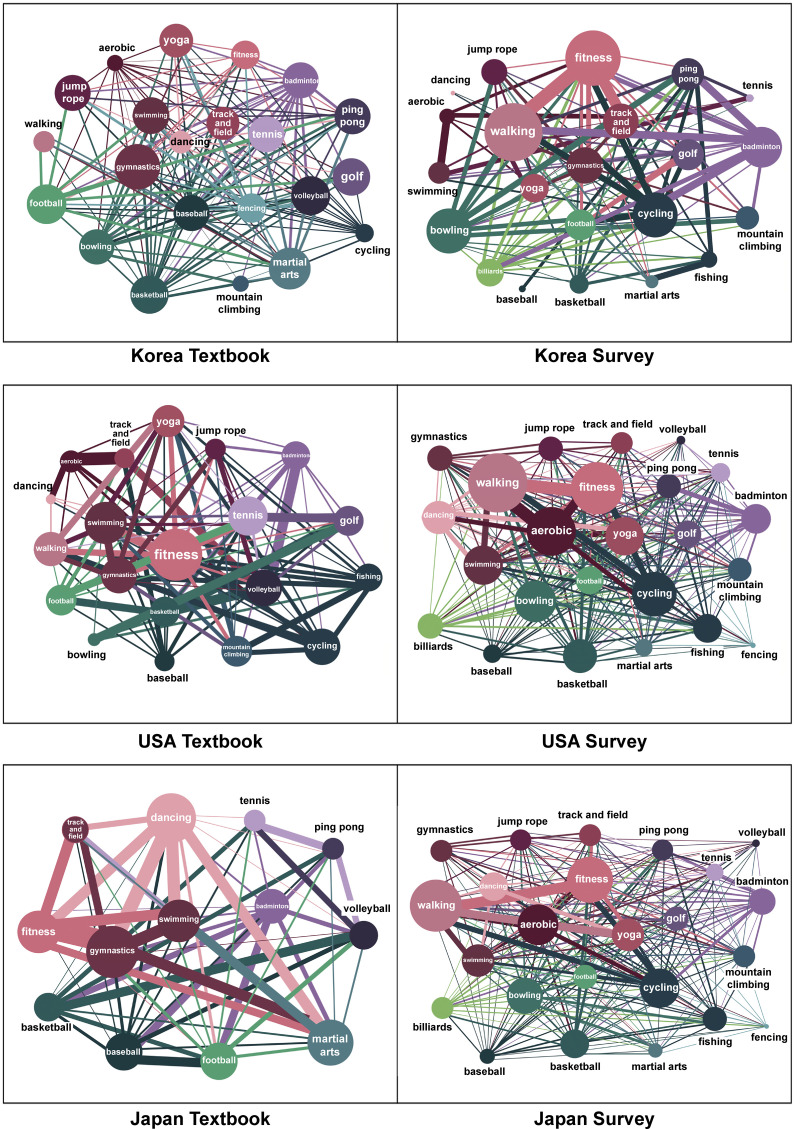
Textbooks and survey (actual physical activity).

Education Systems do not influence the sports activities of adults. Of course, this notion does not signify that there is no use in learning sports that are not enjoyed in everyday life. “The K-12 Physical Education System” consists of curricula regarding the effects of the educational process of students’ physical and psychological developments [37–40]. Accordingly, there is an aspect of the system that intentionally organizes and teaches sports that are difficult to be enjoyed in actual life. It is, however, discovered that there is no relation between the K-12 Physical Education System and actual situations for the activities. This disassociation further suggests that the sports dealt in “The K-12 Physical Education System” were selected more carefully and on scientific grounds (Fig 2).

### SNS is different from the actual physical activity

**Fig 3:**
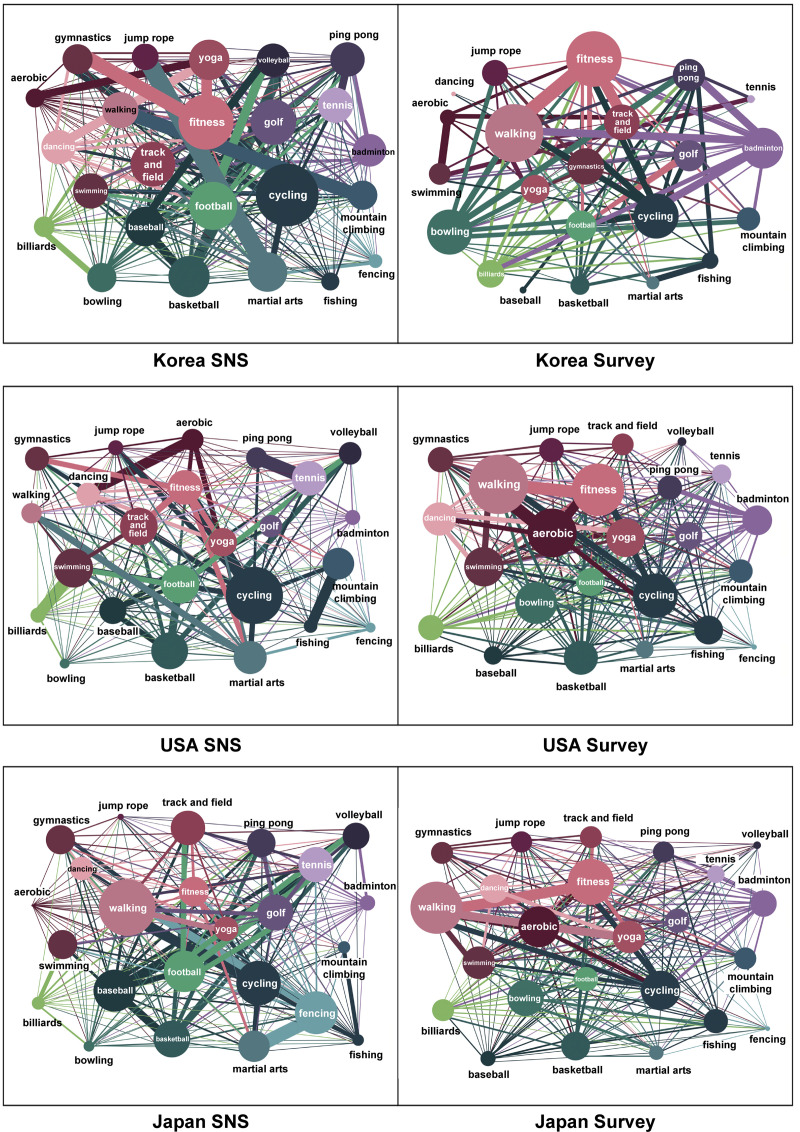
SNS and survey (actual physical activity).

The results of the surveys on actual physical activities and the search for activities-related posts on the Internet are highlighted in Fig 3 and Table 8. As shown above, the differences in Korea, the USA, and Japan are all recognized. Node overlaps of Korea and USA show low rates of similarity, presenting 0.219*** and 0.254***, respectively. In the case of Japan, noticeable similarity cannot be determined with a value of 0.114. The gap between SNS posts and the results of the survey on actual physical activities is thought to be a result of the tendency of SNS activities to boast about one’s activities to acquaintances or unspecified individuals [41]. Therefore, “walking” was not frequently posted in the SNS because it is not thought to have much ability to boast about. On the other hand, cycling and fitness appeared the most in the SNS activities. This occurrence is due to the characteristic of SNS, which can accumulate many reactions to the posts that are fancy and worthy of attention [42]. Hence, the difference between SNS and actual physical activity cannot easily be enjoyed in real life; however, posts that boast about privileges tend dominate.

**Table 8:** Network overlaps (SNS vs. survey).

*X*	*Y*	Edge Overlaps (Jaccard’s coefficient)	Node Overlaps (Pearson’s Correlation coefficient)
Korean SNS	Korea survey	0.138718944	0.2192403***
English SNS	USA survey	0.25946794	0.2542677***
Japanese SNS	Japan survey	0.246101785	0.1143671

* *p* < .05, ** *p* < .01

*** *p* < .001

### Sport classification is rarely correlated in the three fields

**Fig 4:**
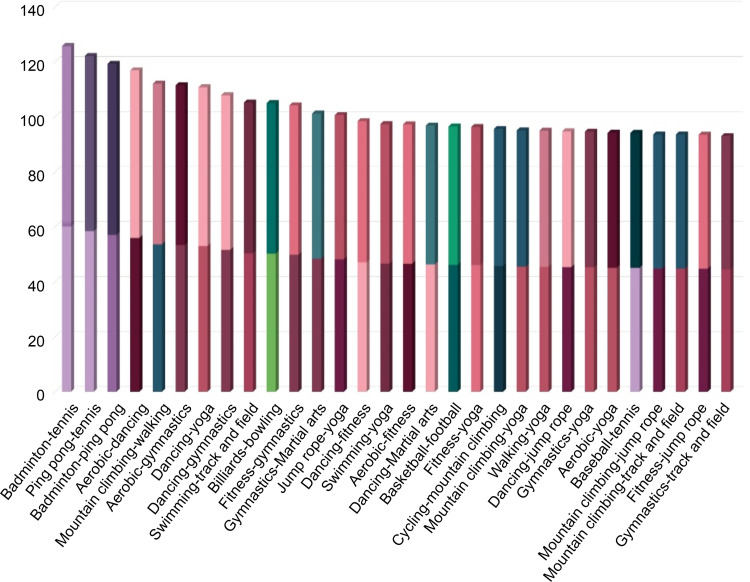
Sport classification—Top 30.

The correlation of Sport Classification is shown in Fig 4. The two sports with the highest similarity are tennis and badminton. While the ranking of the connection between the two is measured as 1 in the sports types, it is measured as low as 204 (Korea), 20 (USA), and 39 (Japan) in textbooks. Besides, it is also very low in the surveys, as in 123 (Korea), 141 (USA), and 139 (Japan). Lastly, the results of the SNS activities were 27 (Korea), 55 (USA), and 113 (Japan). Consequently, the correlations of Sport Classification are considered to have no similarities with all the studied fields, such as educational activities, actual physical activities, and SNS activities (Table 9). The similarities between sports types classified by the expert group were confirmed by measuring various characteristics of the sports types. Thus, ping pong and tennis have a similar relationship, as do tennis and badminton, but the results from the three fields were different. Therefore, it may seem that the similarities between the sports types have no special meaning. However, as the connections quantify the similarities between sports types, it is meaningful to select sports with no similarities for a curriculum that encourages various physical activities.

**Table 9:** Ranking of pairs (Top 30—Sport classification).

Sport Type		Sport Classification	Textbook			Survey			SNS		
			KO	US	JP	KO	US	JP	KO	US	JP
Badminton	Tennis	1	204	20	39	123	141	139	27	55	113
Ping-pong	Tennis	2	204	108	17	58	104	66	13	**1**	95
Badminton	Ping-pong	3	204	108	39	**6**	14	15	19	49	89
Aerobic	Dancing	4	204	15	64	123	**9**	**9**	15	**5**	136
Mountain	Walking	5	204	30	64	44	101	94	**3**	**9**	12
Climbing											
Aerobics	Gymnastics	6	203	108	64	97	154	156	142	51	150
Dancing	Yoga	7	170	108	64	123	12	11	**8**	21	47
Dancing	Gymnastics	8	151	108	**2**	123	96	112	50	101	27
Swimming	Track and	9	38	108	19	123	248	232	60	30	87
	Field										
Billiards	Bowling	10	204	108	64	85	30	34	17	52	83
Fitness	Gymnastics	11	93	11	**1**	42	90	76	**2**	14	51
Gymnastics	Martial	12	**5**	108	**6**	93	209	214	91	81	86
	Arts										
Jumprope	Yoga	13	36	108	64	43	85	80	222	89	211
Dancing	Fitness	14	158	106	**7**	123	32	44	30	40	56
Swimming	Yoga	15	41	24	64	123	27	30	89	119	164
Aerobic	Fitness	16	142	49	64	37	**3**	**3**	53	28	179
Dancing	Martial	17	121	108	**8**	123	178	180	159	103	157
	Arts										
Basketball	Football	18	**3**	**8**	26	36	25	21	31	**6**	97
Fitness	Yoga	19	91	28	64	71	11	13	**5**	18	14
Cycling	Mountain	20	204	13	64	123	140	143	75	26	118
	Climbing										
Mountain	Yoga	21	204	24	64	91	157	118	194	112	198
Climbing											
Walking	Yoga	22	41	14	64	20	**4**	**4**	158	111	**7**
Dancing	Jumprope	23	182	108	64	123	107	115	166	199	211
Gymnastics	Yoga	24	40	35	64	23	62	63	95	36	99
Aerobic	Yoga	25	155	69	64	81	**6**	**6**	11	11	178
Baseball	Tennis	26	**7**	36	43	123	227	178	57	46	**8**
Mountain	Jumprope	27	204	75	64	30	98	81	254	144	264
Climbing											
Mountain	Track and Field	28	155	108	64	107	212	168	111	41	140
Climbing											
Fitness	Jumprope	29	102	50	64	104	124	127	149	31	203
Gymnastics	Track and field	30	**4**	108	15	30	44	35	139	83	107

### Top linked sports types

**Fig 5:**
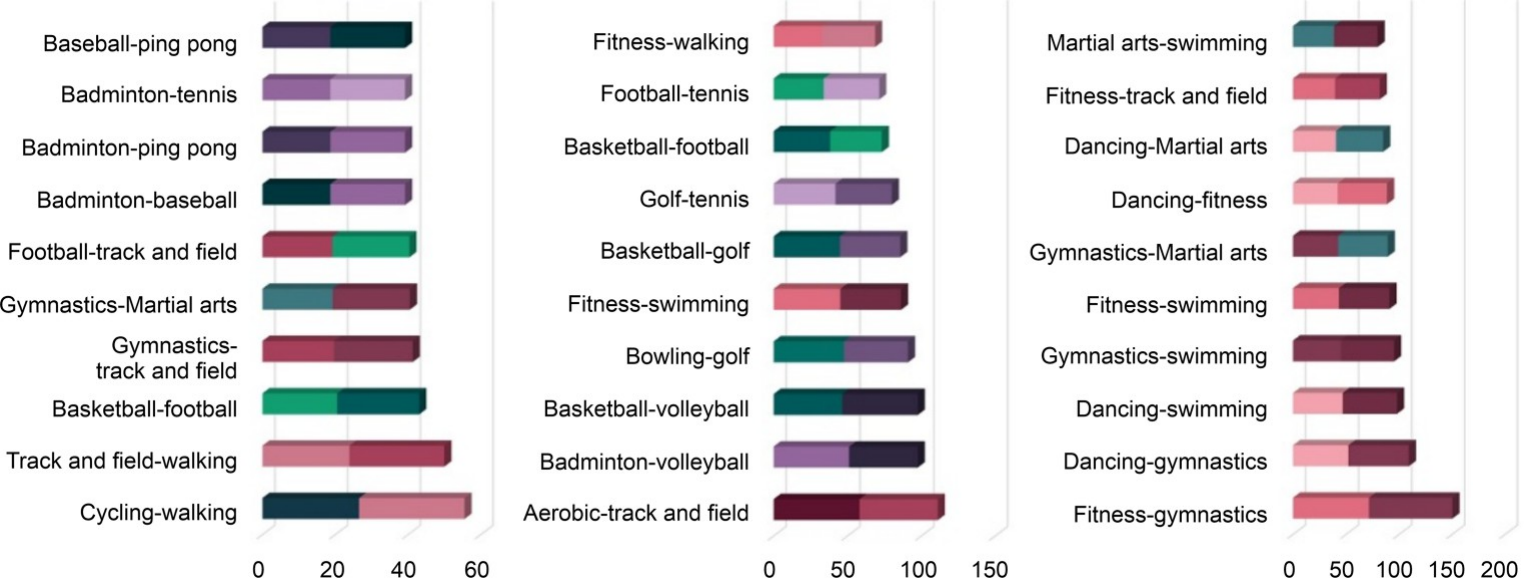
The K-12 Physical Education System Survey, SNS activity—Top 10 (Korea, USA, Japan).

The sports that present the highest relations in each field–“The K-12 Physical Education System”, “Survey (actual physical activity),” and “SNS activities” are seen as follows. First, the high relations in educational activities are as illustrated in Fig 5. Sports with high relations are shown equally in basic sports, such as walking, track and field, fitness, and gymnastics in Korea, the USA, and Japan. Looking into the distinct features of each country, it is noticeable that martial arts are practiced in both Korea and Japan, but not in the USA. According to a study analyzing the perception of martial arts, individuals in Korea and Japan are reported to engage in martial arts for their safety and physical and mental discipline. In particular, as the positive influences on adolescence have been recognized, many adolescents appear to be avid practitioners of martial arts [43]. Martial arts, however, have been popularized in the USA as being centered on people with special goals, such as attaining a certain degree of physical fitness [44]. Therefore, it seems that this knowledge is not applied to educational activities owing to the belief that the practice can transform adolescents’ bodies into weapons and lead to serious violence.

**Fig 6:**
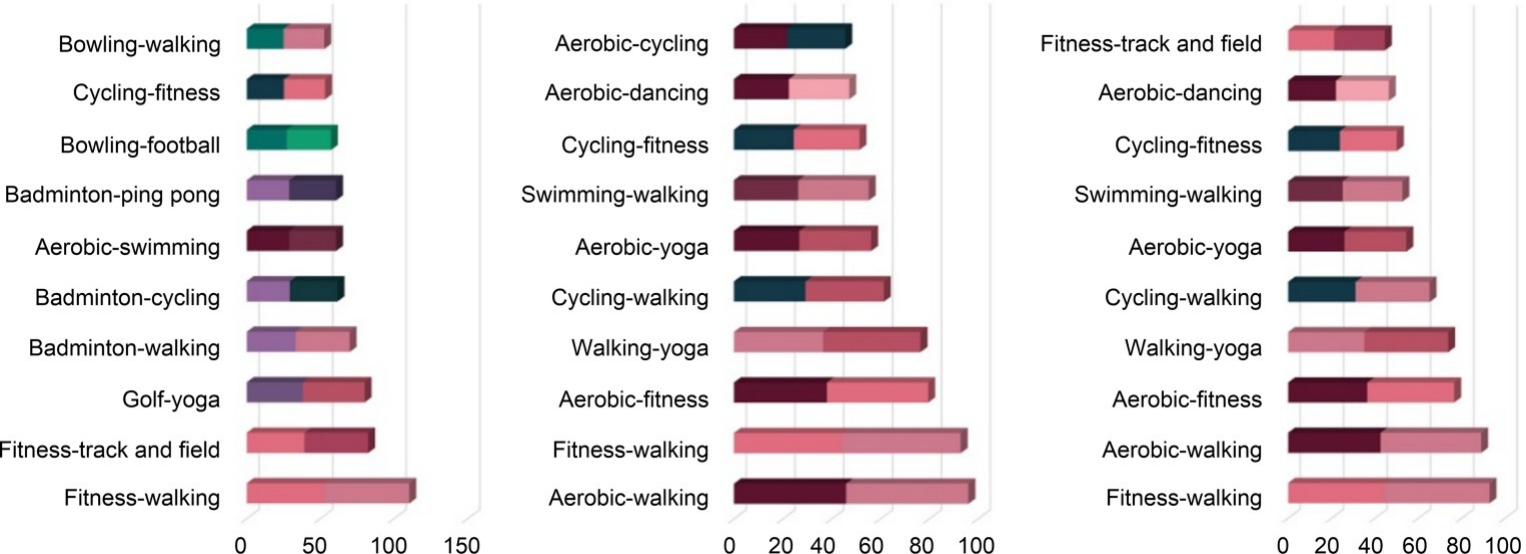
Survey (actual physical activity)—Top 10 (Korea, USA, Japan).

Secondly, the relations indicated in actual physical activities are depicted in Fig 6. Walking indicates high relations in all the countries, as shown in the actual physical activities. Basic sports, such as fitness and aerobics, are shown equally in Korea, the USA, and Japan. Also, sports such as yoga appear to be practiced universally and have been adopted as actual physical activities. In Korea, the correlation between golf and yoga is high. That is to say, people enjoying yoga enjoy golf. Notably, the sports facilities, including 467 outdoor and 10,335 indoor golf courses, are equipped to allow people to enjoy golf easily [45]. Interestingly, yoga is enjoyed by many women in Korea [46]. Since yoga can be easily accessed therefore it is highly favored by Korean women, and many people seem to be enjoying golf and yoga simultaneously.

**Fig 7:**
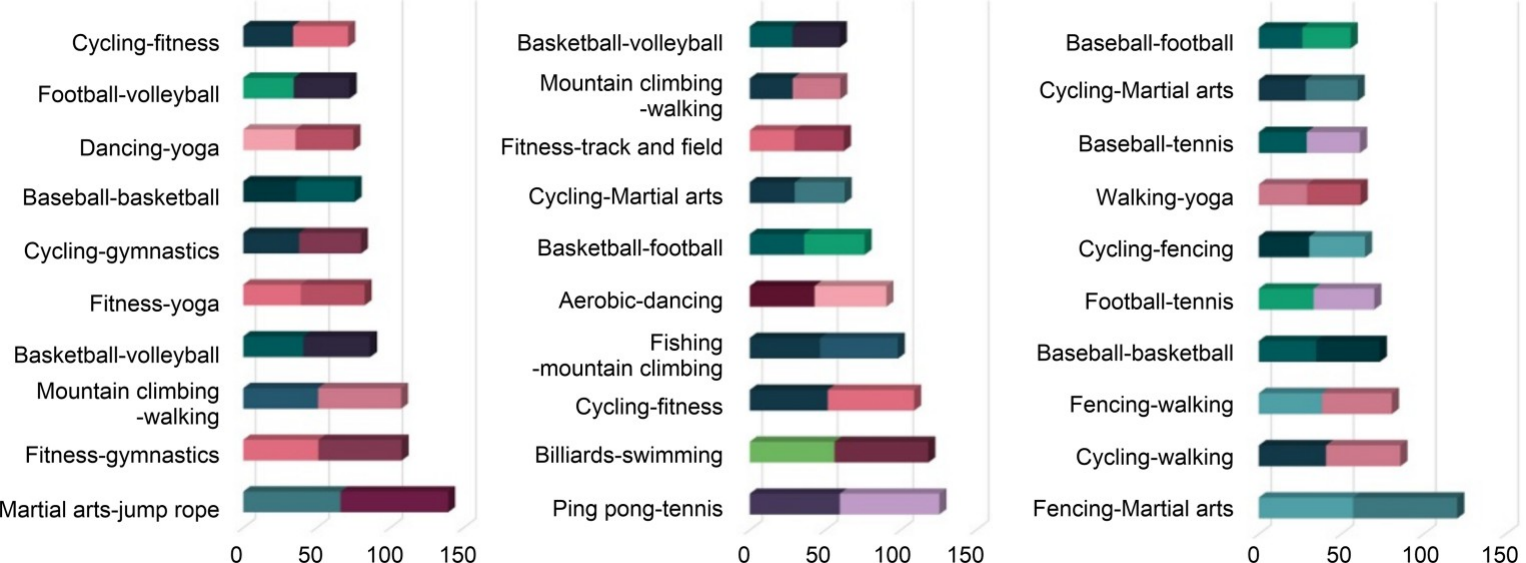
SNS activities—Top 10 (Korea, USA, Japan).

Thirdly, the correlations shown in SNS activities are exhibited in Fig 7. The highest correlations are shown for martial arts and jump rope in Korea, ping-pong and tennis in the USA, and fencing and martial arts in Japan as per the SNS activities of each country. Regarding the connections between sports in each country, the highly connected sports are martial arts and jump rope, with fitness and gymnastics ranking second, and mountain climbing and walking placing third. This finding suggests that a high connection can be seen between highly related sports. However, in the case of the USA, while the highest connection is between ping-pong and tennis, the correlation between billiards and swimming ranking second could not be found. Furthermore, despite the ranking that gives third place to cycling and fitness, fishing and mountain climbing, two seemingly unrelated sports, were given fourth place. The connections between two otherwise unconnected sports are also ranked in the networks of SNS activities in the USA. The result for hiking and fishing in the United States may reflect accessibility issues because these are activities that sometimes require approval for access to participate. In contrast, in Korea and Japan, these sports do not require a permit. Lastly, fencing and martial arts are ranked in its highest position, cycling and walking in the second, and fencing and walking as third highest in Japan. For Japan, it seems that the sports that appear to be highly correlated are ranked high, and the third rank is given to football and tennis. Consequently, the connections between different kinds of sports do not have consistent influences on SNS activities. Popular sports, such as basketball, baseball, and football also appeared evenly in the SNS of each country. The result seems to be attributed to the fact that the popularity of sports is related to the SNS activities of the fans [47]. Ultimately, it can be explained that SNS activities are related to both the connections between sports and their popularity.

**Fig 8:**
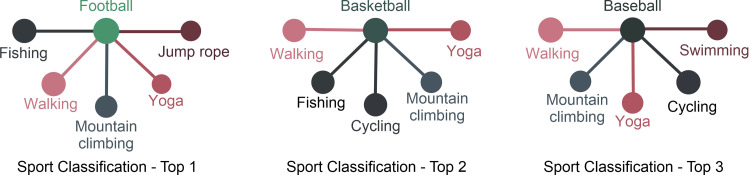
Highly connected sports (Sport classification).

Finally, the graphs display the top linked sports in the four fields. As shown in Fig 8, the popular sports (i.e., football, basketball, and baseball) are highly connected to various athletics. This result was produced only by survey questionnaires concerning the characteristics of sports types and proved that the existing popular sports have substantial interconnections. This outcome can explain why popular sports today have gained mass popularity. That is to say that popular sports (i.e., football, basketball, and baseball) are considered to have been at the center of the 24 sports not because of external factors like marketing, but because of their unique characteristics. The 41 items selected by the expert group represent the characteristics of each sport. We investigated various characteristics, such as using a large stadium, the participation of several players, and using a goal post. These characteristics appear to differing degrees in popular and unpopular events. A detailed comparison of the characteristics of popular and unpopular sports can elucidate the reasons behind popularity and lack of popularity. Therefore, the comparison can help determine the factors that lead unpopular sports to fail in gaining popularity among the public. Thorough study of the relationship between sports characteristics can allow inference of the reason unpopular sports do not attract public attention. Through this, we can determine approaches to increase public interest in unpopular sports.

**Fig 9:**
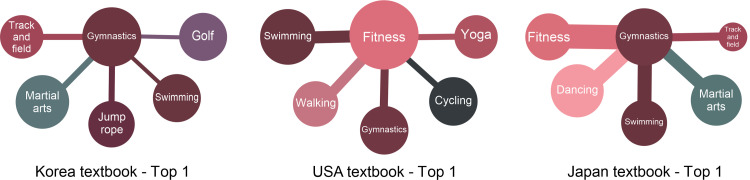
Highly connected sports (Textbook).

As shown in Fig 9, gymnastics (Korea), fitness (USA), and gymnastics (Japan) take the highest place in terms of their connections with other sports regarding educational activities. That is, the principle that emphasizes basic sports seems to be reflected in the curriculum [48]. Looking further into the differences between the countries, martial arts and golf are highly connected in Korea. On the contrary, martial arts and dancing display a high connection in Japan, while swimming and cycling are highly connected in the USA. Korea and Japan have common features in track and field, martial arts, and swimming, while Korea includes golf and jump rope, and Japan includes dancing and fitness. Although they share common features that place great significance on basic sports, there are differences in the sports connected to nationally. It seems that cultural variations have been reflected in the process of organizing the curricula.

**Fig 10:**
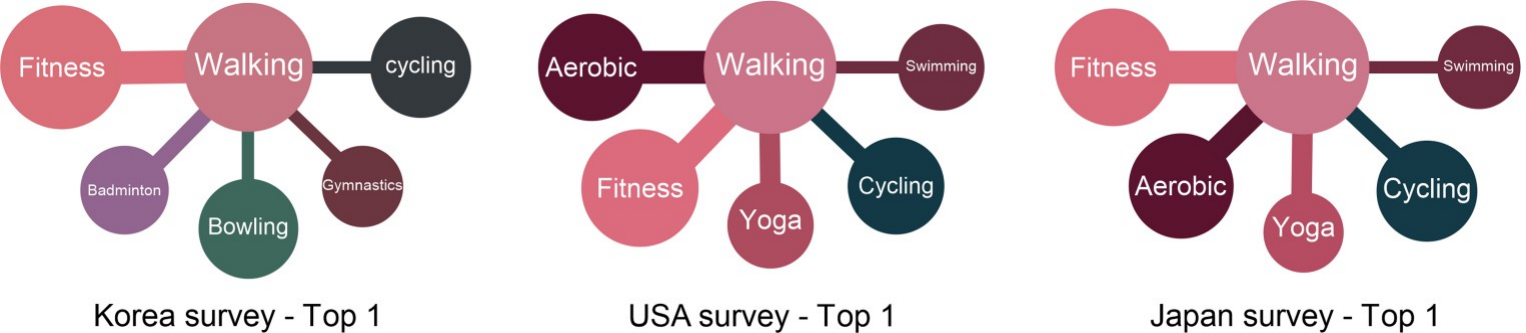
Highly connected sports (Survey).

Through the survey (actual physical activities), walking displays the highest connection with other sports (Fig 10). Based on the result that fitness, gymnastics, aerobic, and cycling are highly connected, the basic sports are seen to be mostly played as actual physical activities. There is a noticeable result that swimming is preferred in Japan and the USA, but badminton and bowling replace them in Korea. The result appears to be attributed to the fact that Korea has only 1% of swimming pools on a national average, which is very scarce compared to Japan, which reports a penetration rate of swimming pools ranging between 90%–98% for primary schools in each region [49]. It reminds us of the necessity to expand sports facilities to popularize sports.

**Fig 11:**
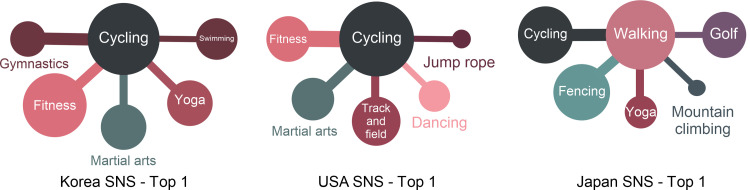
Highly connected sports (SNS).

As seen in Fig 11, cycling in Korea and the USA and walking in Japan are highly connected to SNS activities. SNS activities are different from actual physical activities in that the users mainly post pictures of sports with spectacular subjects such as cycling, martial arts, and fitness. This phenomenon is because SNS activity can reveal ostentatiousness [50]. To upload posts solely to show off makes it equally necessary to take pictures that attract the attention of the masses. Since cycling is an outdoor sport, it seems to have high connections. What is special here is that walking has the most connections on SNS in Japan. It seems to reflect the cultural characteristics of Japan, which values everyday triviality.

### Comparison with sport classification

The degree of connection between sports types was compared among the four fields. Textbooks, surveys, and SNS were compared based on the sport classification. Table 10 shows the ranking of sports types in the four fields.

**Table 10:** Ranking of four fields (Sport types linked).

Sport Type	Sport Classification	Textbook			Survey			SNS		
		KO	US	JP	KO	US	JP	KO	US	JP
Football	1	3	10	6	10	12	11	3	4	5
Basketball	2	5	11	10	16	8	8	6	6	6
Baseball	3	9	18	7	21	21	21	8	11	4
Walking	4	19	7	14	1	1	1	10	18	1
Cycling	5	20	5	14	3	4	4	1	1	2
Volleyball	6	4	4	9	23	23	23	15	15	15
Fishing	7	23	14	14	17	11	12	24	22	21
Billiards	8	23	22	14	12	14	14	21	21	19
Fencing	9	16	22	14	24	24	24	23	24	3
Golf	10	6	12	14	9	13	13	4	14	7
Badminton	11	10	13	8	5	10	10	16	20	18
Mountain Climbing	12	22	9	14	14	18	17	19	9	20
Bowling	13	13	19	14	4	5	5	18	23	22
Ping Pong	14	11	22	13	8	16	18	13	17	14
Tennis	15	12	3	12	20	20	20	12	7	8
Swimming	16	7	2	5	15	6	6	11	3	12
Martial arts	17	2	21	3	19	22	22	9	8	10
Yoga	18	14	8	14	11	7	7	7	10	16
Aerobic	19	21	15	14	18	3	3	22	16	24
Jump Rope	20	8	16	14	13	17	19	20	19	23
Dancing	21	18	20	2	22	9	9	14	13	17
Fitness	22	17	1	4	2	2	2	2	2	11
Track and Field	23	15	17	11	7	19	16	5	5	9
Gymnastics	24	1	6	1	6	15	15	17	12	13

According to the results, football, basketball, and baseball are located in a high degree of connectivity, while fitness, track and field, and gymnastics are located in a low degree of connectivity. Football, basketball, and baseball are the most popular sports. Fitness, track and field, and fitness are basic sporting events. There are many different sports; some are popular and the others are less popular. This evidence can be attributed to characteristics, such as strength, stamina, speed, density, aggression, and team spirit [51]. The ranking of the connection diagrams presented here denotes where each sport is located. Therefore, we compared the links shown in sports classification and the line in textbooks, surveys, and SNS as follows. The number of sports connections was measured in the order of sports types. The graph of the sport classification is prepared on the y-axis and textbooks, surveys, and SNS on the x-axis.

**Fig 12:**
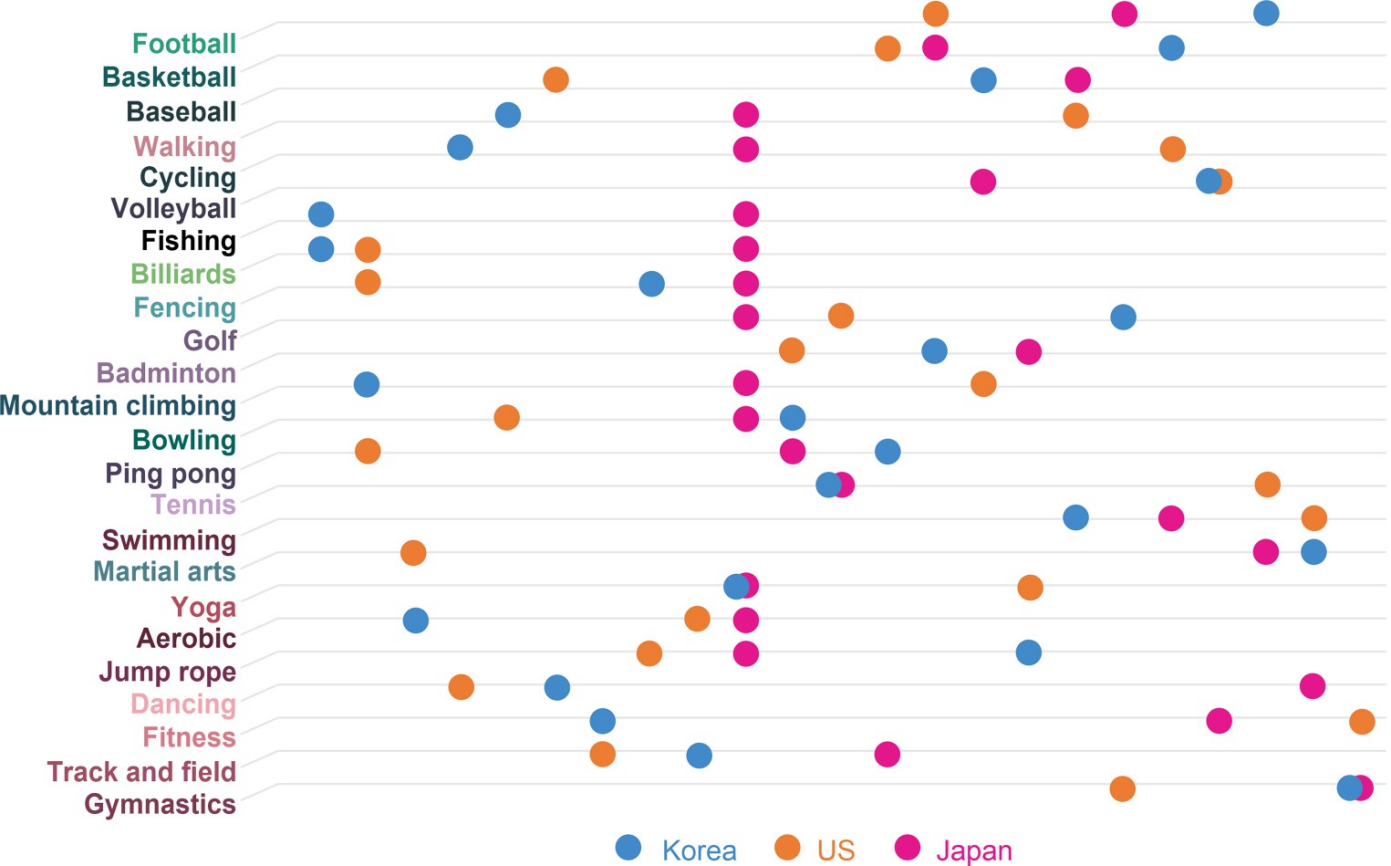
Comparison with textbooks.

The results show that the rankings of sports covered in textbooks in Korea, the United States, and Japan makes it difficult to find common features (Fig 12). The system that contains the contents of the physical education curriculum is similar in general, but it has national characteristics. Korea has a national-level curriculum, textbooks, and teacher guidance, while the United States presents instruction manuals for teachers in core regional-level curriculum (Lee, 2017 [35]). Japan offers specific details of physical education classes for students by grade (Lee and Koo, 2011 [36]). The results are believed to have been led by the fact that each country has different physical education policies.

**Fig 13:**
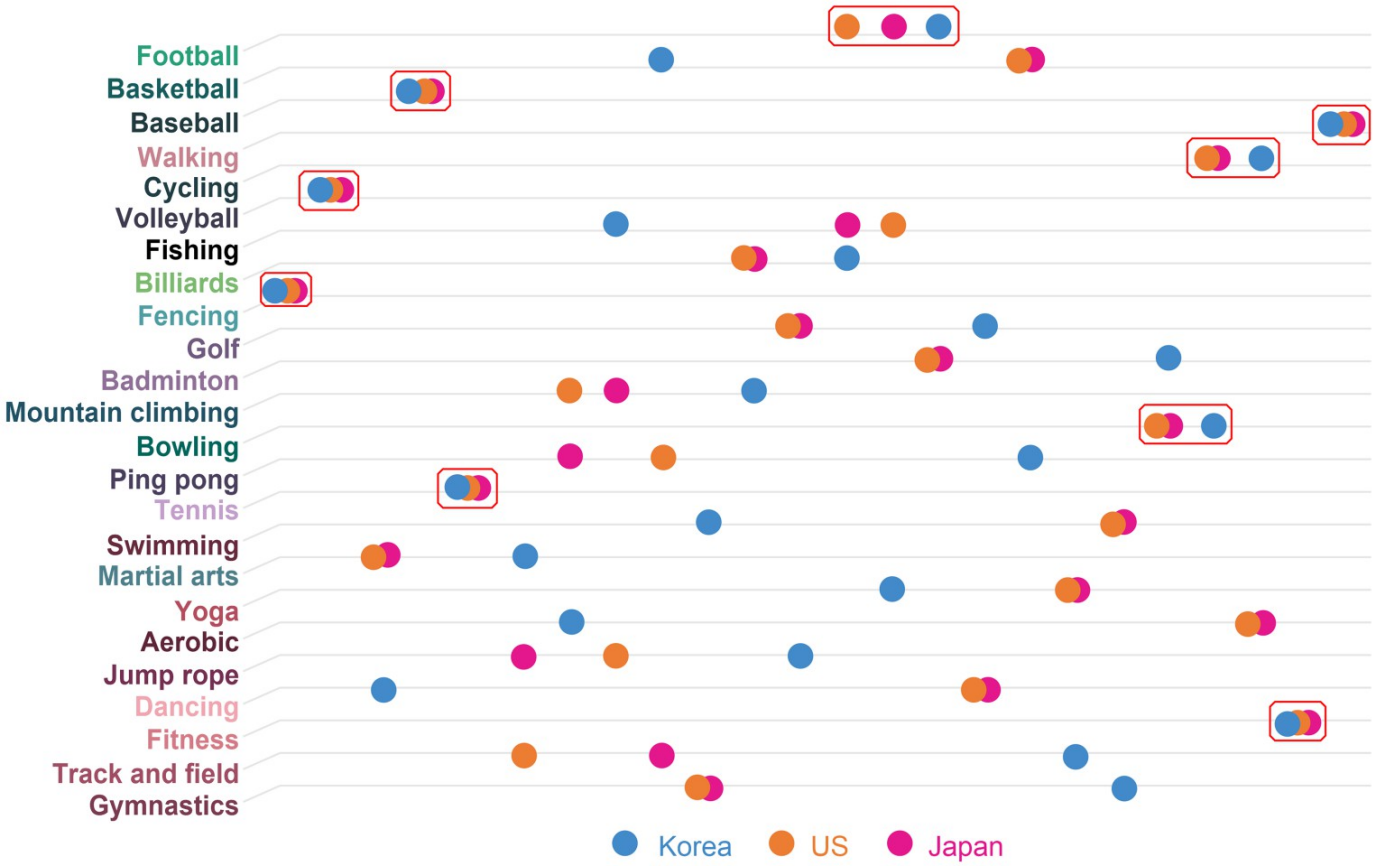
Comparison with survey.

A comparison of the survey and sports classification is shown in Fig 13. The results of this survey verify the state of sports that people actually play. The findings highlight that football, walking, cycling, and fitness are found in high places. Volleyball, billiards, bowling, and tennis were commonly in low positions. According to a study that analyzed the correlation between demands for sports and national income, basketball, soccer, volleyball, track and field, and martial arts have little correlation with national income. On the other hand, there are also reports that team sports, walking, and fishing are highly correlated with income [52]. As of 2018, the GNI of Korea, the U.S., and Japan have a difference of 30,600 in Korea, 62,850 in the U.S., and 41,340 in Japan. Nevertheless, it appears that no particular difference was detected in physical activity.

**Fig 14:**
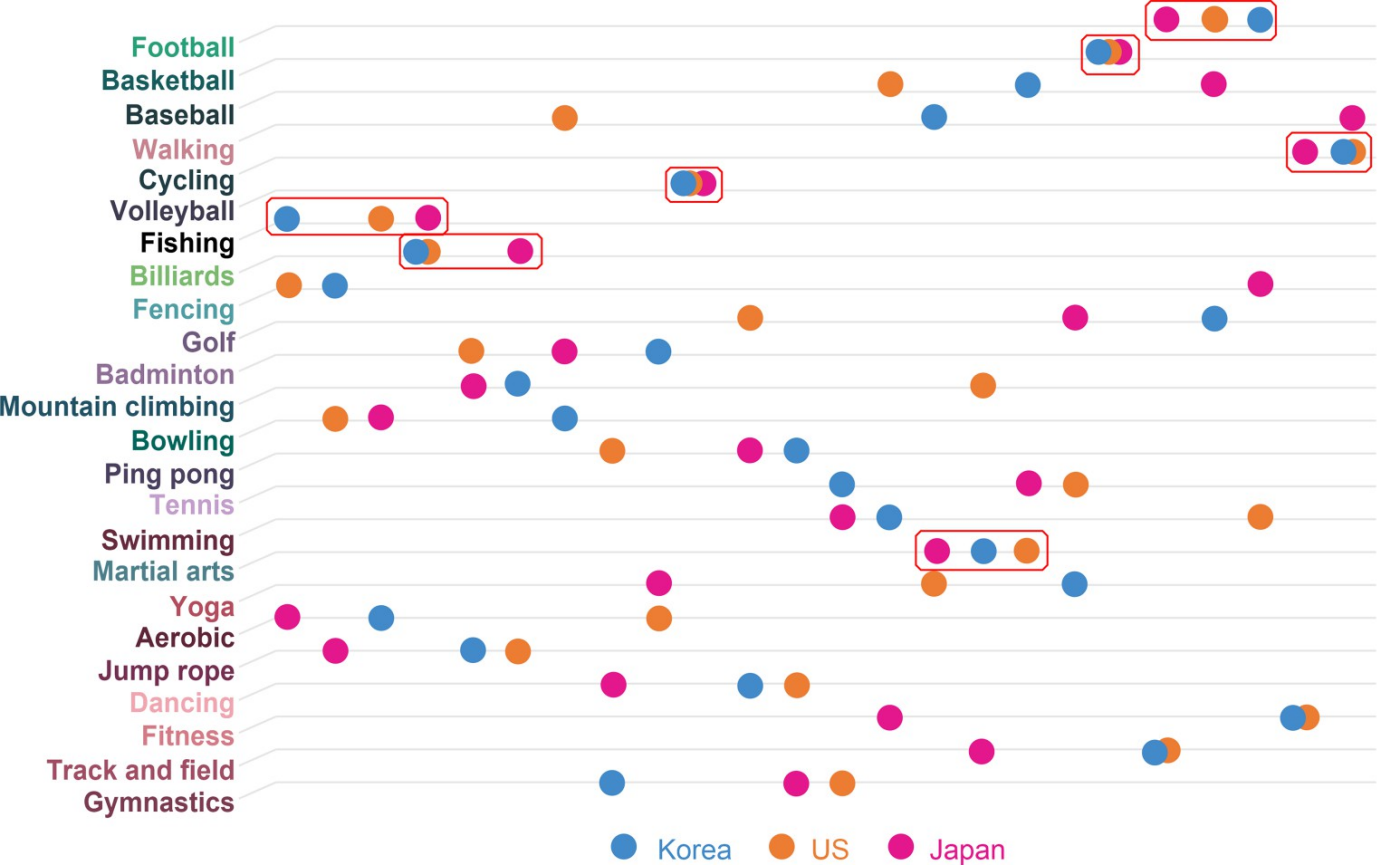
Comparison with SNS.

A comparison of SNS and sport classifications is shown in Fig 14. SNS results show the status of sports types that people want to show off. According to the results, all three countries show the same position for football, baseball, cycling, fishing, and martial arts.

Instagram users collected in this study’s post-SNS phase reported various motivations, such as social interaction, archiving, self-satisfaction, escape, and peeping income [53]. The study found that events, such as football, basketball, cycling, and martial arts were highly displayed in SNS activities among the three countries. The frequent display of football and basketball on SNS seems to be caused by the fact that they have strong fan bases. Cycling and martial arts are shown to have a high degree of connection due to self-examination and self-satisfaction income [54].

The ranking of the degree of connection according to the characteristics of each sport type has the following meaning. The top sports types of connection are the most popular sports, and the bottom sports types are unpopular sports. Comparing rankings according to the classification of sport types with textbooks, surveys, and social networking sites can be used to identify the location of sport types.

The academic distinction between popular and unpopular sports is not clear, but popular sports can be considered as sports that have been established as professional, activated by their sports club members, or have developed sports infrastructure as per the national base expansion. Moreover, winning medals at various Olympic Games, World Cup tournaments, and Asian Games is meaningful, but the public’s perception of unpopular sports is considerably low. This belief is a hindrance to the balanced development of sports, which requires a balanced selection of sports.

Textbooks, surveys, SNS, and comparison of Sport Classification have the following meanings. The diversity among textbooks covers is clearly evident. The results of the survey confirmed the actual activities of sports. These results can be used as basic data to infer the position of a sport type by identifying sport classifications. It can also be used as a reference to select sports to be covered in textbooks.

## Conclusion

This study models the sports types as well as “The K-12 Physical Education System,” “Survey (actual physical activities),” and “SNS activities” in Korea, USA, and Japan through the sports networks and analyses them. As a result, four implications are summed up as follows.

First, what was learned from the curriculum and actual physical activities examined through the surveys varies. Second, there are distinct differences between the three countries based on the K-12 Physical Education System, Survey (actual physical activities), and SNS posting activities. Third, SNS posting activities and actual physical activities vary. Fourth, the sports networks on Sport Classification, the K-12 Physical Education System, Survey (actual physical activity), and SNS activities differ significantly.

As identified in the connections between different types of sports, the K-12 physical education system does not influence the actual physical activities. If schools are expected to provide various experiences from different sports, the current curriculum can be appropriate since it already handles many types of sports. However, there can be an opposite opinion suggesting that the curriculum focusing on basic sports is more appropriate in a limited educational environment than in sports that are never played in adulthood. The notable point here is that the students should learn sports to maintain their physical and mental health. To achieve this goal, it is necessary to think more carefully about the structure of the curriculum on the K-12 physical education system, which balances between giving experiences of various sports and teaching the basic sports.

## References

[1] Chicago Tribune. 25 Years Ago, Michael Jordan Played for the White Sox against the Cubs at Wrigley Field—and got 2 hits. 2019 April 7. Available from: https://www.chicagotribune.com/sports/white-sox/ct-spt-white-sox-michael-jordan-cubs-wrigley-field-20190407-story.html.

[2] Korean Sport & Olympic Committee. 100 Years of Sport Korea. 2020. Available from: https://portal.sports.or.kr/info/webzineView.do?boardSeq=HSA0068444.

[3] essentiallysports. Watch: When Rafael Nadal And Cristiano Ronaldo Played Foot-Tennis. 2020a.

[4] essentiallysports. Rafael Nadal’s Love for Real Madrid and Football. 2020b.

[5] The New York Times. Tiger Woods and Serena Williams Are Friends With a Comeback Connection. 2018.

[6] Jones CD, Hollenhorst SJ, Perna F, Selin S. Validation of the flow theory in an on-site whitewater kayaking setting. J Leis Res. 2000; 32(2): 247.

[7] Madrigal R. Investigating an evolving leisure experience: Antecedents and consequences of spectator affect during a live sporting event. J Leis Res. 2003; 35(1): 23–48.

[8] Ogden DC, Hilt ML. Collective Identity and Basketball: An Explanation for the Decreasing Number of African-Americans on America’s Baseball Diamonds. J Leis Res. 2003; 35(2): 213–227.

[9] Pondé MP, Santana VS. Participation in leisure activities: Is it a protective factor for women’s mental health? J Leis Res. 2000; 32(4): 457.

[10] KISS. An analysis of international sports policy trends. KISS: Seoul. 2015.

[11] Ahn B-W, Cho E-Y. The verification of relationship model among self-efficacy, enjoyment factor, flow experience, and university life satisfaction by general physical class participants. J Korean Soc Wellness. 2015; 10(3): 97–107.

[12] Ministry of Culture, Sports and Tourism (Korea). A survey on the participation in sports activities in Korea. Ministry of Culture, Sports and Tourism. Seoul. 2018.

[13] Japan Sports Agency. Public Opinion Survey on the Implementation of Sports, etc. 2019. https://www.mext.go.jp/sports/b_menu/toukei/chousa04/sports/1415963_00001.htm.

[14] Physical Activity Council. The Physical Activity Council’s annual study tracking sports, fitness, and recreation participation in the U.S., Physical Activity Council., Jupiter. 2019.

[15] Ministry of Education (Korea). Physical Education Course. Ministry of Education. Seoul. 2015.

[16] Ministry of Education, Culture, Sports, Science and Technology (Japan). Elementary School Physical Education Course. Ministry of Education, Culture, Sports, Science and Technology. Tokyo. 2017a.

[17] Ministry of Education, Culture, Sports, Science and Technology (Japan). Middle School Physical Education Course. Ministry of Education, Culture, Sports, Science and Technology. Tokyo. 2017b.

[18] Ministry of Education, Culture, Sports, Science and Technology (Japan). High School Courses Physical Education Course. Ministry of Education, Culture, Sports, Science and Technology. Tokyo. 2017c.

[19] Couturier L, Stevie C, Shirley HH. National Standards & Grade-Level Outcomes for K–12 Physical Education. SHAPE America—Society of Health and Physical Educators: Reston. 2014.

[20] Scarpa S, Nart A. Influences of perceived sport competence on physical activity enjoyment in early adolescents. Soc Behav Pers. 2012; 40(2): 203–204.

[21] Tammelin T, Näyhä S, Laitinen J, Rintamäki H, Järvelin M-R. Physical activity and social status in adolescence as predictors of physical inactivity in adulthood. Prev Med. 2003; 37(4): 375–381. doi: 10.1016/s0091-7435(03)00162-2 14507496

[22] Diez-Roux AV. Neighborhoods and health: where are we and where do we go from here? Rev Epidemiol Sante Publique. 2007; 55(1): 13–21. doi: 10.1016/j.respe.2006.12.003 17320330 PMC1906739

[23] Dhar SK, Hoch SJ, Kumar N. Affective category management depends on the role of the category. J Retail. 2001; 77(2): 165–185.

[24] Chakravarthi N, Neslin SA, Sen SK. Promotional elasticities and category characteristics. J Mark. 1996; 60(2): 17–30.

[25] Carter ML, Valenti SS, Goldberg RF. Perception of sports photographs: a multidimensional scaling analysis. Percept Mot Skills. 2001; 92(3): 643–652. doi: 10.2466/pms.2001.92.3.643 11453187

[26] Levine DM, Levine PM. Nonmetric Multidimensional Scaling and Hierarchical Clustering: Procedures for the Investigation of the Perception of Sports. Res Q Am Assoc Health Phys Educ Recreat. 1977; 48: 341–348. 267975

[27] Gelsing LE. Innovation and the Development of Industrial Networks. In: Lundvall B-Å, editors. National Systems of Innovation: Towards a Theory of Innovation and Interactive Learning. Frances Pinter Publishers Ltd; 1991.

[28] Knoke D, Kuklinski J. Network Analysis. Sage: London; 1982.

[29] Bullmore ET, Sporns O. Complex brain networks: graph theoretical analysis of structural and functional systems. Nat Rev Neurosci. 2009; 10(3): 186–198. doi: 10.1038/nrn2575 19190637

[30] Haznagy A, Fi I, London A, Nemeth T. Complex network analysis of public transportation networks: A comprehensive study. International Conference on Models & Technologies for Intelligent Transportation Systems (MT-ITS). 2015. 371–378.

[31] Kim Yong-wook, Han Jinyoung, Kyungtae Jang. Modeling and Analyzing Sports Networks revealed through K-12 Physical Education Systems: Case Studies of Korea, Japan, and the USA. Proceedings of icSPORTS 2019. 2019(1).

[32] Yong-Yeol A, Ahnert SE, Bagrow JP, Barabási A-L. Flavor network and the principles of food pairing. Sci Rep. 2011. doi: 10.1038/srep00196 22355711 PMC3240947

[33] Sohn E, Noh K-R, Lee B, Kwon O-J. Bibliometric network analysis and visualization of research and development trends in precision medicine. 2018 IEEE/ACM International Conference on Advances in Social Networks Analysis and Mining (ASONAM). 2018. 727–730.

[34] Wäsche H. Interorganizational cooperation in sport tourism: A social network analysis. Sport Manag Rev. 2015; 18(4): 542–554.

[35] Lee K-M. Comparative analysis of elementary school’s physical activity related health in Korea and Utah state. J Educ Stud. 2017; 54(4): 1–15.

[36] Lee D-Y, Koo B-J. A comparative study of elementary school physical education curriculum between Korea and Japan. Korean Journal of the Japan Education. 2011; 16(1): 87–103. http://www.earticle.net.ssl.access.hanyang.ac.kr/Journal/Detail/340.

[37] Johnson PB, Updyke WF, Stolberg DC, Schaefer M. Physical education: A problem-solving approach to health and fitness: A textbook for men and women. New York, NY: Holt, Rinehart and Winston; 1966.

[38] Johnson DJ, Harageones EG. A health fitness course in secondary physical education: The Florida experience. In: Pate RR, Hohn RC, editors. Health and fitness through physical education. Champaign, IL: Human Kinetics; 1994. pp. 165–172.

[39] Penney D, Jess M. Physical education and physically active lives: A lifelong approach to curriculum development. Sport Educ Soc. 2004; 9(2): 269–287.

[40] Tinning R, Fitzclarence L. Postmodern youth culture and the crisis in Australian secondary school physical education. Quest. 1992; 44: 287–303.

[41] Sheldon P, Bryant K. Instagram: Motives for its use and relationship to narcissism and contextual age. Comput Human Behav. 2016; 58: 89–97.

[42] Carpenter CJ. Narcissism on Facebook: Self-promotional and anti-social behavior. Pers Individ Dif. 2012; 52(4): 482–486.

[43] Kim J-T, Kim S-T. The relationship between social support, positive psychological capital and school adjustment of multicultural family adolescent students who participate in martial-art sports activity. J Martial Arts. 2018; 12(3): 61–82.

[44] Jennings G, Cynarski WJ. Martial arts in postcolonial times: Local theories for local contexts. J Martial Arts Anthropol. 2019; 19(3): 11–23.

[45] STATISTICS KOREA. Physical activity facilities. 2020 http://index.go.kr/potal/stts/idxMain/selectPoSttsIdxMainPrint.do?idx_cd=1664&board_cd=INDX_001.

[46] Kang J-Y, Lee S-Y. A study on the deepening process of women’s participation in Yoga: The grounded theory approach. Korean J Sport. 2017; 15(3): 577–591.

[47] Phua J. Use of social networking sites by sports fans: Implications for the creation and maintenance of social capital. J Sports Media. 2012; 7(1): 109–132.

[48] Housner LD. Innovation and change in physical education. In: Silverman S, Ennis C, editors. Student learning in physical education. Human Kinetics, USA:IL; 1996. pp. 367–389.

[49] The DONG-A ILBO. Only 1% of “Swimming Elementary School”… Japan is 90% public. 2019. Available from: http://www.donga.com/news/article/all/20191114/98348948/1.

[50] Kim S-M, Hwang H-J, Lee H. A phenomenological study on experiences of social network (SNS) leisure activity: Focusing on women in their twenties. Int J Tour Sci. 2018; 42(3): 11–31.

[51] Apostolou M, Athanasiou M. I want to watch this! An evolutionary perspective on the popularity of sports. Psychol Top. 2016; 25(2): 281–297.

[52] Lee KJ, Song MG. The correlation between per capita GDP and the demand of sports items and the income elasticities of sports items`demand: Based on an international comparison data. Korean J Sport Sci. 2014; 25(4): 713–725.

[53] Lee E, Lee J-A, Moon J-h, Sung Y. Pictures Speak louder than words: Motivations for using Instagram. Cyberpsychol Behav Soc Netw. 2015; 18(9): 552–556. doi: 10.1089/cyber.2015.0157 26348817

[54] Ross AS, Zappavigna M. My sport, my perspectives: Intersubjectivity in cyclist instagram posts. Discourse Context Media. 2020; 34(2020): 100327.

